# Non-Equal Contributions of Different Elements and Atomic Bonds to the Strength and Deformability of a Multicomponent Metallic Glass Zr_47_Cu_46_Al_7_

**DOI:** 10.3390/molecules29215005

**Published:** 2024-10-22

**Authors:** Donghua Xu, Olivia Gordon, Muyang Ye, Lei Chen, Tittaya Thaiyanurak, Zhengming Wang

**Affiliations:** 1 Materials Science Program, Oregon State University, Corvallis, OR 97331, USA; 2 School of Mechanical, Industrial and Manufacturing Engineering, Oregon State University, Corvallis, OR 97331, USA; 3 School of Arts and Sciences, Atlanta Metropolitan State College, Atlanta, GA 30310, USA; 4 Department of Computer Science, University of Southern California, Los Angeles, CA 90007, USA; muyangye@usc.edu

**Keywords:** metallic glass, multicomponent alloy, atomic bonds, material strength, material deformation

## Abstract

Multicomponent metallic glasses (MGs) are a fascinating class of advanced alloys known for their exceptional properties such as limit-approaching strength, high hardness and corrosion resistance, and near-net-shape castability. One important question regarding these materials that remains unanswered is how the different elements and atomic bonds within them control their strength and deformability. Here, we present a detailed visual and statistical analysis of the behaviors of various elements and atomic bonds in the Zr_47_Cu_46_Al_7_ (at%) MG during a uniaxial tensile test (in the z-direction) simulated using molecular dynamics. Specifically, we investigate the identities of atoms undergoing significant shear strain, and the averaged bond lengths, projected z-lengths, and z-angles (angles with respect to the z-direction) of all the atomic bonds as functions of increasing strain. We show that, prior to yielding, the Zr element and the intermediate (Zr-Zr, Cu-Al) and stronger (Zr-Al, Zr-Cu) bonds dominate the elastic deformation and strength, while the Cu and Al elements and the weaker Al-Al and Cu-Cu bonds contribute more to the highly localized shear transformation. The significant reconstruction, as signified by the cessation of bond-length increment and bond-angle decrement, of the intermediate and the stronger bonds triggers yielding of the material. After yielding, all the elements and bonds participate in the plastic deformation while the stronger bonds contribute more to the residual strength and the ultimate (fracture) strain. The results provide new insights into the atomic mechanisms underlying the mechanical behavior of multicomponent MGs, and may assist in the future design of MG compositions towards better combination of strength and deformability.

## 1. Introduction

Metallic glasses (MGs) are solid metallic materials that do not contain any crystals, in stark contrast to conventional metals/alloys (including high-entropy alloys) which consist of numerous crystal grains. The amorphous or glassy structure endows MGs with a host of fascinating properties, for example, limit-approaching strength, high elastic strain limit, exceptional resilience, high wear- and corrosion-resistance, near-net-shape castability, and thermoplastic processability at temperatures far below the melting point [1,2,3,4,5,6]. The first MG was reported in the Au-Si binary system in 1960 which could only be fabricated in the form of thin foils (~10 μm thickness) by quenching the molten alloy at a very high cooling rate (~10^5^ to 10^6^ K/s) on a specially designed apparatus (splat quencher) [7]. Nowadays, various MGs have been discovered in a broad range of alloy systems, e.g., Zr-based [8,9,10], Cu-based [11,12,13], Hf-based [14,15,16], and Ti-based [17,18], which can form bulk products with the smallest dimension exceeding 1 mm, using more typical mold casting methods (cooling rates ~10^3^ K/s or lower). With a few notable exceptions of binary alloys (e.g, Cu-Zr [12,19,20], Cu-Hf [19,21], Ca-Al [22]), most contemporary bulk MGs are multicomponent, containing three or more elements with a range of atomic sizes and electronegativities [8,9,10,11,12,13,14,15,16,17,18,23,24,25,26,27,28].

Being a relatively new class of materials, MGs are not yet widely used in commercial products or engineering applications. Many questions about these novel materials remain to be answered. One key question is how the different elements and atomic bonds inside a multicomponent MG control the material’s mechanical behavior. This question holds both fundamental and practical significance. Fundamentally, it is known that under a uniaxial load, a MG initially deforms elastically with some distributed shear transformation zones (STZs, tiny clusters of atoms) undergoing a small amount of localized plastic deformation, and upon yielding, planar features termed “shear bands” (thin layers of sheared atoms) occur and allow the material to deform plastically (to a quite limited degree usually) until fracture. However, which elements and atomic bonds are more responsible for the elastic deformation and strength, and which others for the plastic deformation (STZs and shear bands), are not well understood. From a practical point of view, understanding the roles of the different elements and atomic bonds could aid in designing new MG compositions to address the known weakness of MGs: their limited plasticity (at ambient temperature).

There have been some experimental efforts to tackle these questions through in situ mechanical tests on synchrotron beamlines [29,30,31]. However, due to the disordered atomic structure, the information provided by the diffraction data is quite limited and projecting such data into the rich context of elements and atomic bonds often requires empirical mathematical models and, sometimes, omission of certain elements or bonds.

Here, we use atomistic simulation, specifically, molecular dynamics (MD), combined with atomistic visualization (also known as computational microscopy) and statistics, to directly examine the behaviors of the different elements and atomic bonds in an exemplary multicomponent MG, Zr_47_Cu_46_Al_7_, during a uniaxial tensile test. This composition is selected due to its own status as a bulk MG with relatively good glass-forming ability, its close relationship with several landmark binary and multicomponent (Cu_46_Zr_54_ [12,19], Cu_46_Zr_42_Al_7_Y_5_ [12,19] and Cu_46_Zr_33.5_Hf_13.5_Al_7_ [11]) bulk MGs, and the availability of an interatomic potential (EAM—Embedded Atom Method—type) that describes the interactions among its constituent elements. Based on the visualization and the statistical data (e.g., average bond lengths and bond angles), we will draw conclusions about the contributions of different elements and atomic bonds to the strength and deformability of the multicomponent MG.

## 2. Results and Discussion

The RDF is one statistical tool to characterize the atomic structure and atomic-neighbor distance in a material/system. It is generally defined to be the atomic number density within a radial distance of r to r+dr from an average atom, normalized by the overall atomic number density in the system, i.e., $g\left(r\right)=\frac{\rho (r)}{\rho_{ideal}}=\frac{dN(r)}{\rho_{ideal}4\pi r^{2}dr}$, where $\rho_{ideal}=N_{tot}/V_{tot}$, dN(r) is the count of atoms located inside the spherical shell between r and r+dr, $N_{tot}$ is the total number of atoms in the material, and $V_{tot}$ is the sample volume. The RDF, i.e., $g\left(r\right)$ vs. r, curve consists of a set of peaks, representing the different (1st, 2nd, 3rd, …) coordination shells (or, neighbor distances). For a crystalline material, the peaks on the RDF curve appear as sharp spikes because the crystal structure has well-defined neighbor distances. In contrast, for disordered materials such as liquids or glasses, the RDF peaks appear as diffuse maxima, due to the randomness of atomic positions and interatomic distances. The RDF can be computed for all atoms together, or for a selected type of elemental pairs only (e.g., Cu-Cu, Zr-Cu). While the former case, i.e., the total RDF, reflects the overall atomic structure and atomic-neighbor distance, irrespective of elemental types, the latter, i.e., a partial RDF, provides additional information about the atomic distribution/distance for a specific type of elemental pairs.

**Figure 1:**
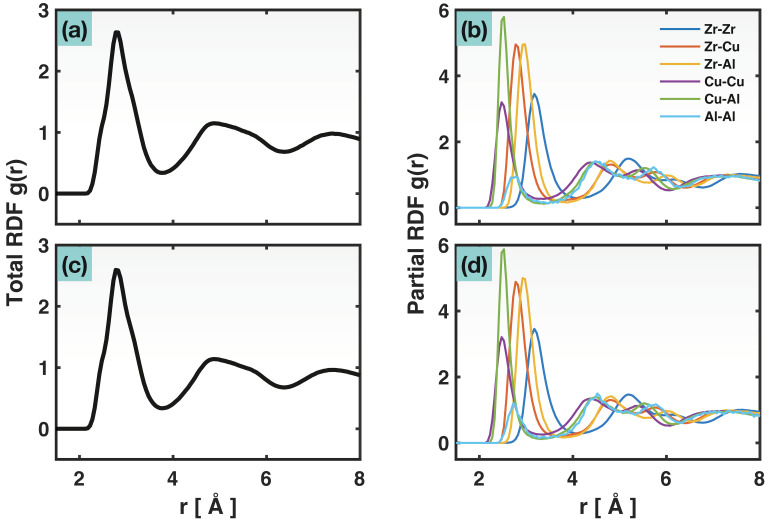
Total radial distribution function (**a**,**c**) and partial radial distribution functions (**b**,**d**), at an overall strain of 0 (**a**,**b**) and 30% (**c**,**d**).

Figure 1a shows the total RDF of the Zr_47_Cu_46_Al_7_ sample after relaxation at 300 K, before the tensile testing started. The absence of sharp spikes on the RDF attests to the fully glassy nature of the sample. Figure 1b presents the partial RDFs for all the six possible types of elemental pairs, including like pairs, Zr-Zr, Cu-Cu, Al-Al, and unlike pairs, Zr-Cu, Zr-Al, and Cu-Al. As discussed above, the domain of the first peak on a partial RDF is related to the range of the first nearest-neighbor distance of that specific pair type, or in other words, the range of the bond length for that pair type. Therefore, the valley between the first and second peaks on a partial RDF defines a cutoff distance that can be used to numerically search for bonding atoms of the specific pair type. The cutoff distances hence obtained for the six types of bonds are listed in Table 1. It may be tempting, given the cutoff distances in Table 1 and the first-peak positions on the partial RDFs in Figure 1b, to consider one type of bond, e.g., Cu-Cu, to be shorter than another, e.g., Zr-Cu. However, one must keep in mind that each type of bond in the glassy material has a span (range) of bond lengths and there is overlap between the spans for the different bond types.

**Figure 2:**
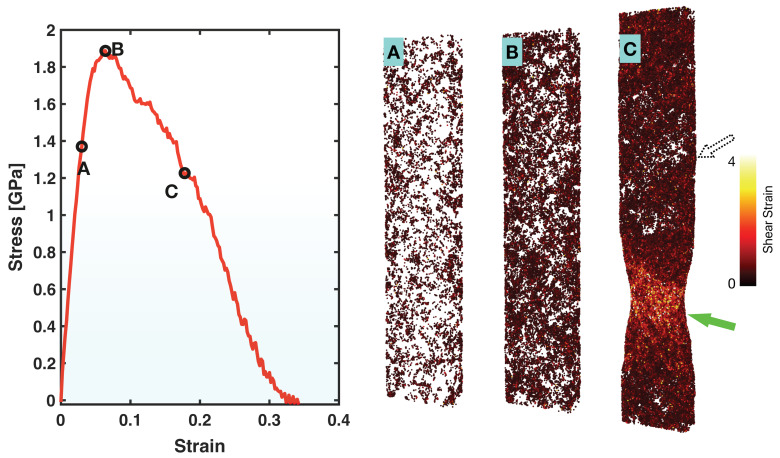
Stress-strain curve (**left**) and maps (**right**) of atoms with relatively large atomic shear strain (>0.3) at the three states, (**A**–**C**), marked on the stress-strain curve. The dotted hollow arrow marks the position of an incompletely formed shear band and the filled arrow marks the main active shear band that causes the overall sample-shape change during post-yielding deformation.

Figure 1c,d present the total and the partial RDFs at a 30% (0.3) nominal strain, a timestep very close to the fracture (32.4% strain) of the sample (see Figure 2 for the stress–strain curve). It should be noted that the RDFs computed by OVITO use the simulation cell volume for $V_{tot}$ which can differ considerably from the sample volume after deformation and sample-shape change. Corrections using the actual sample volume (determined through the “construct surface mesh” modifier in OVITO) have been implemented here. Comparing Figure 1c,d with Figure 1a,b, respectively, there is virtually no discernable change in the total and partial RDFs from the start to the end of the tensile test; in other words, these RDFs are rather insensitive to the strain of the sample. On one hand, this suggests that the same set of cutoff values for defining the various atomic bonds can be used throughout the tensile test. On the other hand, it may seem surprising that the RDF peak positions or peak shapes are unaffected by the fairly large strain and even fracture. One reason for this is that, although RDFs are closely related to the atomic bond lengths, they are not exactly a survey of bond length distribution. Another reason is that the RDFs here are non-directional, related to the bond lengths in all directions. In some synchrotron experiments [29,30,31], two-dimensional X-ray diffraction patterns have been used to derive the total RDF (or another version of it known as pair distribution function G(r)) resolved in the loading direction. However, the challenge in those experiments is the deconvolution of the total RDF into the partial RDFs for different elemental pairs.

The left panel in Figure 2 presents the tensile stress-strain curve obtained here for the Zr_47_Cu_46_Al_7_ MG. The overall shape of the curve is typical among MGs in MD simulations, consisting of an upward nearly straight (mostly elastic) segment at the beginning, a peak in stress (signifying the yielding of the material), and then a downward wavy (plastic) segment. While the maximum stress (yield strength) here, ~1.9 GPa, appears close to what is often observed for Zr- or Cu-based MGs in experiments [11,19,20], the intention of this study is not to reproduce experimental stress-strain curves, but rather to examine the behaviors of the different elements and atomic bonds through the multiple stages of deformation, that is, pre-yielding, (around) yielding, and post-yielding.

The right panel in Figure 2 shows the maps of atoms that have experienced relatively large shear strain (>0.3 here, calculated in reference to the starting atomic configuration) at three representative states, A (pre-yielding), B (around yielding), and C (post-yielding), which are marked on the stress-strain curve. Prior to yielding, large-shear-strained atoms are the centers of STZs that undergo highly localized plastic deformation, even though the overall deformation of the material is mostly elastic. The map for State A shows that the STZs are essentially isolated and well-distributed (albeit with preference for free surfaces in x- and y- directions) prior to yielding. Around yielding, as shown by the map for State B, spatial correlations of STZs have developed in certain planar directions. After yielding, as shown by the map for State C, a shear band fully loaded with large-shear-strained atoms appears and becomes the active center of plastic flow, causing an apparent change in sample shape. The overall deformation stages and the sequential STZ and shear band development presented in Figure 2 are consistent with what is known to date about the general mechanical behavior of MGs.

**Figure 3:**
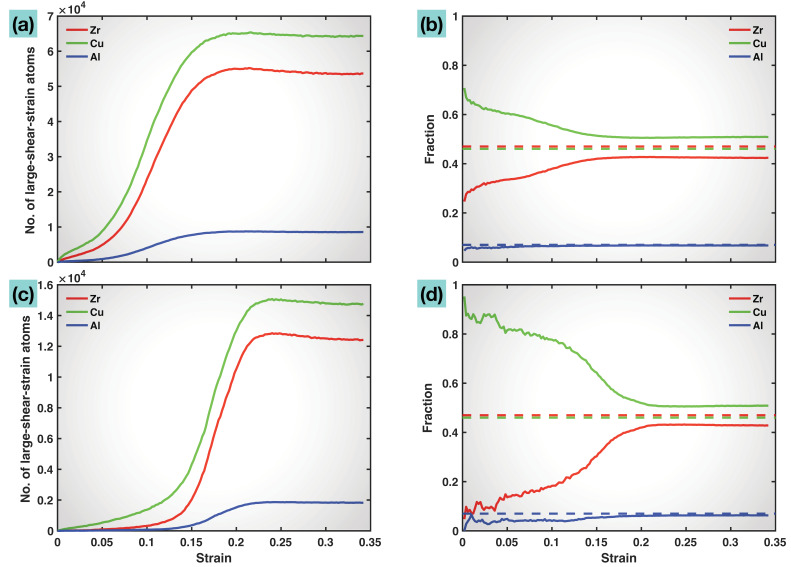
Number of atoms (**a**,**c**) of each element exceeding a threshold atomic shear strain of 0.3 (**a**) and 1.0 (**c**), and the corresponding elemental fraction among the large shear-strained atoms: (**b**) for (**a**), and (**d**) for (**c**). The dashed lines in (**b**,**d**) mark the elemental fractions in the overall material composition.

With the large-shear-strained atoms selected out, their elemental identities can be analyzed. Figure 3a,b plot the numbers (counts) of atoms and the fractions of the different elements among the large-shear-strained group (>0.3 atomic shear strain), as functions of the overall tensile strain. In terms of numbers, all the three elements have more and more atoms participating in the large-shear-strained group as the tensile strain increases, while the number of Cu atoms in the group is always the greatest, followed by Zr and then Al. In terms of fraction, the Cu fraction in the group decreases whereas the Zr and Al fractions both increase with increasing tensile strain. However, the Cu fraction in the group always stays the highest and clearly above the Cu fraction (46%) in the overall material composition. This shows that Cu makes the biggest contribution to the plastic deformation of the multicomponent MG, especially in the pre-yielding stage (in the form of STZs). In contrast, the Zr fraction in the large-shear-strained group is significantly below the Zr fraction (47%) in the material composition, which is also particularly noticeable prior to yielding. The Al fraction in the group is slightly below the Al fraction (7%) in the material composition in the early stage, and later approaches very close to 7%.

Similar trends are observed in Figure 3c,d where a higher threshold of atomic shear strain, 1.0, is used to select the large-shear-strained group of atoms. With this higher selection threshold, the dominance of the Cu fraction and the subordination of the Zr fraction in the group in the early stage of deformation are even more evident. This again affirms that prior to yielding, Cu is the most significant contributor to the localized plastic deformation (STZs), and, opposite this, Zr mainly contributes to the elastic deformation and strength. After yielding, especially in the later stage of deformation (beyond ~0.1 tensile strain), Zr also actively participates in the plastic deformation (mainly in the form of a shear band), working together with Cu and Al to allow the material to continuously change its shape and dimensions. The important contribution of Zr to the elastic deformation and strength will be further substantiated later by the bond visualization and bond length and bond angle statistics.

While Figure 3 clearly shows the relative contributions of the three elements to plastic (or the opposite, elastic) deformation, it does not contain information about atomic bonds. Cu atoms, for example, are bonded by other Cu atoms, Zr atoms, and/or Al atoms. Are these bonds contributing equally to the plastic or elastic deformation? To show the behaviors of atomic bonds, we have recorded six MD movies (with a 0.02 increment in the overall tensile strain between two frames) for Zr-Zr, Zr-Cu, Zr-Al, Cu-Cu, Cu-Al, and Al-Al bonds, respectively, focusing on an area in the lower half of the sample, near where the shear band develops, and within a central slice of 1 nm thickness in the x (smallest) dimension. These movies are provided as Supplementary Materials S1–S6, in the same order as the above list.

Before discussing the bond behaviors, we divide the bonds into three categories: weak bonds (Al-Al and Cu-Cu), intermediate bonds (Cu-Al and Zr-Zr), and strong bonds (Zr-Al and Zr-Cu), based on the cohesive energies (for like bonds) [32] and enthalpy of mixing (for unlike bonds) [33] listed in Table 2. Strictly speaking, the cohesive energies and enthalpy of mixing from the literature are not rigorous measures of the bond strengths in an MG, because, for each type of bond, there is a span of bond lengths (hence bond energies) due to the disordered structure. However, as will be shown later, this categorization aligns with the bond behaviors under increasing tensile strain.

**Figure 4:**
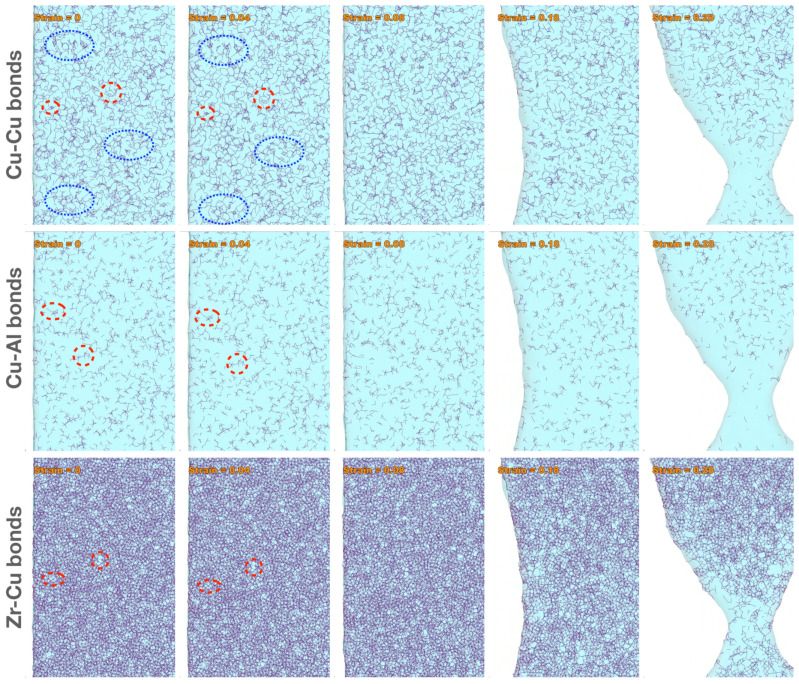
Snapshots (enlarged and cropped for clarity) from MD movies (Supplementary Materials) showing the behaviors of Cu-Cu (**top row**), Cu-Al (**middle row**), and Zr-Cu (**bottom row**) bonds across five levels of tensile strain, 0, 0.04, 0.08, 0.18, and 0.28. The red dashed ellipses outline examples of corresponding bonds. The blue dotted ellipses outline examples of reconstructed bonds.

Selected snapshots (enlarged and cropped for clarity) from the movies for Cu-Cu (Movie S4), Cu-Al (Movie S5), and Zr-Cu (Movie S2), representing the weak, intermediate, and strong bond categories, respectively, are shown in Figure 4. From both the movies and the snapshots in Figure 4, one can observe the bonds being stretched (and some shortened), rotated, and reconstructed (some broken and some newly established) during the tensile test. To guide the eyes, two pairs of red dashed ellipses are added to the snapshots for each of the representative bond types in Figure 4 at the tensile strain of 0 and 0.04 (pre-yielding), which outline examples of corresponding features (bonds) between the two states. While most Cu-Al and Zr-Cu bonds and some Cu-Cu bonds are only stretched and rotated from 0 to 0.04 tensile strain, there are evident bond reconstructions in various places for the Cu-Cu bonds at this early stage of deformation, some examples being outlined by the blue dotted ellipses in Figure 4. This shows that the weak Cu-Cu (as well as Al-Al) bonds are the main contributor to the pre-yielding localized plastic deformation (STZs), while the intermediate and strong bonds contribute more to the elastic deformation and the strength of the material.

By the tensile strain of 0.08 (just past yielding), evident bond reconstructions are also recognized at different locations for the Cu-Al and Zr-Cu bonds. This implies that the yielding is likely triggered by the reconstructions of the intermediate and strong bonds. Further after yielding (see snapshots at the tensile strain of 0.18 and 0.28 in Figure 4, or the Movies S1–S6), significant bond reconstructions continue for all the bonds, particularly in the vicinity of the active shear band, while the bonds far away from the shear band are still being stretched and rotated. By the tensile strain of 0.28, severe bond breakage is evident for all the bonds around the shear band, although the intermediate Zr-Zr bonds (see Movie S1) and strong Zr-Cu and Zr-Al (Movie S3) bonds are still serving as a linkage between the two parts of the sample on the opposite sides of the shear band before they break apart. This implies that the intermediate and strong bonds are the main contributor to the residual strength and the limit of plastic deformation (i.e., the fracture strain) after yielding.

**Figure 5:**
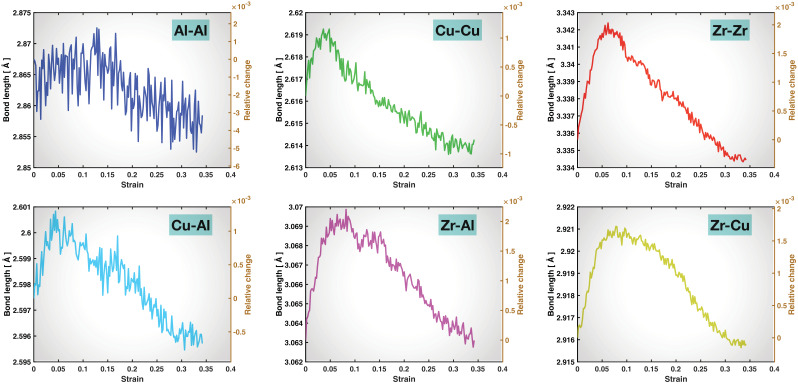
Averaged bond length as a function of overall tensile strain for the six types of bonds. The right y-axis in each panel provides the relative change in the averaged bond length with respect to the start (0 strain) of the tensile test.

**Figure 6:**
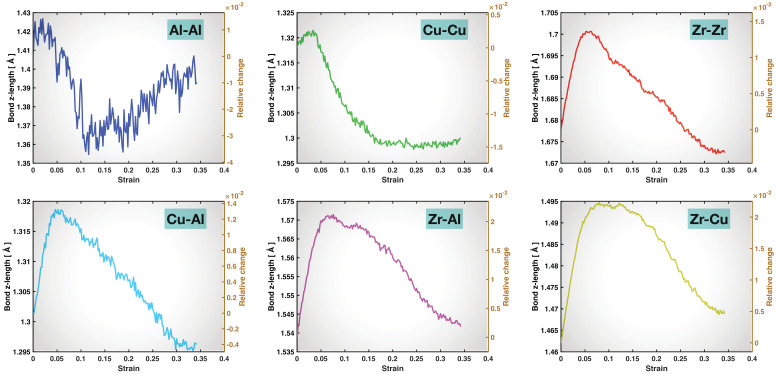
Averaged bond z-length as a function of overall tensile strain for the six types of bonds. The right y-axis in each panel provides the relative change in the averaged bond z-length with respect to the start (0 strain) of the tensile test.

**Figure 7:**
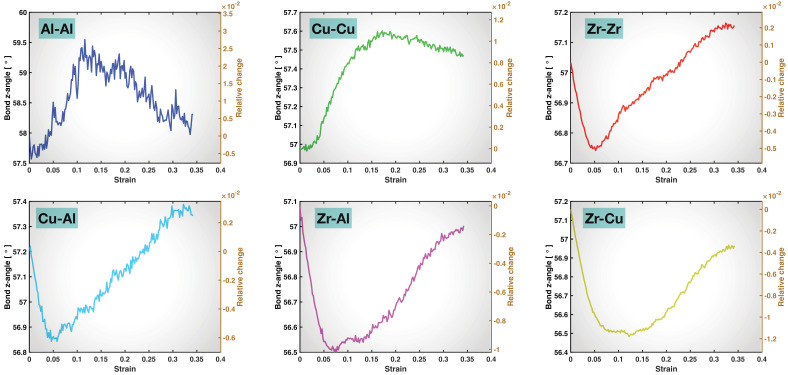
Averaged bond z-angle as a function of overall tensile strain for the six types of bonds. The right y-axis in each panel provides the relative change in the averaged bond z-angle with respect to the start (0 strain) of the tensile test.

To show the bond behaviors more quantitatively, we have computed the averaged bond length, bond z-length, and bond z-angle for each type of bond as functions of the overall tensile strain, which are plotted in Figure 5, Figure 6 and Figure 7, respectively. As shown in Figure 5, the averaged bond length initially increases and then decreases for all the six types of bonds. The initial increase in the averaged bond length is expected as the bonds are overall stretched. The later decrease is also expected, because the bonds are then reconstructed and the newly formed bonds are more relaxed and less stretched. It is not very clear when the averaged bond length starts to drop for the Al-Al and Cu-Al bonds due to the relatively high level of noise which is partly caused by the low percentage of Al in the alloy composition and partly by bond shrinkage in x- and y-directions (related to bond rotation). However, it is evident that the drop in the averaged bond length starts earlier for the Cu-Cu bonds than for Zr-Zr, Zr-Al, and Zr-Cu. Indeed, it starts before 0.05 tensile strain for the Cu-Cu bonds, which is clearly earlier than the apparent yielding of the material (~0.06 to 0.07 tensile strain) shown by the stress-strain curve in Figure 2. This is consistent with the evident reconstruction of Cu-Cu bonds in the early stage of pre-yielding deformation revealed by the visualization discussed above. For the Zr-Zr, Zr-Al, and Zr-Cu bonds, the drop in the averaged bond length coincides more in timing with the apparent yielding of the material. Another interesting feature to point out here is that the relative change in the averaged bond length with respect to the start of the tensile test during the bond-stretching stage, as denoted by the right-side y-axis in the plots of Figure 5, is on the order of 0.1 to 0.2% for all the six bond types, which is very small but significant.

Since the tensile load is along the z-direction, the bonds are mainly stretched in the z-direction, while being rotated towards (more parallel to) the z-axis, prior to the material’s yielding, or more appropriately, prior to the reconstruction of each bond type (which could be termed as “individual bond yielding”). We can expect to see the trend of bond stretching (and rotation) and its turning point more clearly by projecting the bond length (and bond angle) onto the z-axis. As shown in Figure 6, the averaged bond z-length exhibits a brief and relatively small increment for Al-Al and Cu-Cu bonds, up to ~1% and 0.3% relative change with respect to the start, respectively, and then starts to drop at ~0.04 tensile strain, which, again, is clearly earlier than the apparent yielding of the material. In contrast, the intermediate bonds, Zr-Zr and Cu-Al, sustain stretching up to ~1.3% relative change in the averaged bond z-length, and the strong bonds, Zr-Al and Zr-Cu, up to ~2.1% and 2.2% relative change, respectively. And, for all the latter four bond types, the bond z-length increment continues until very close to the material’s yielding, again indicating the correlation between the reconstruction of these intermediate and strong bonds and the overall material yielding. The magnitudes of the relative changes in the averaged bond z-lengths of all the bond types (including the weak bonds) during the bond stretching stage here are notably higher than those in the non-projected overall bond lengths in Figure 5, as expected based on the loading direction.

Some more detailed features can also be recognized in Figure 6. One is that, after the material’s yielding (~0.06 to 0.07 tensile strain), the averaged bond z-length drops more slowly for the strong Zr-Al and Zr-Cu bonds than for the intermediate Zr-Zr and Cu-Al bonds, and even more slowly than for the weak Al-Al and Cu-Cu bonds. This can also be seen in Figure 5, although the lower noise level here in Figure 6 makes it more evident. This implies that the strong bonds, with assistance from the intermediate bonds, are providing the post-yielding residual strength and allowing the material to continuously deform until final fracture. Another interesting feature from Figure 6 is that the averaged bond z-length stops dropping in the late stage of post-yielding deformation for the weak bonds, and even rises again in the case of Al-Al bonds. This is because these weak bonds are severely destructed/depleted in the vicinity of the shear band in this stage, more so than the intermediate and strong bonds. The remaining weak bonds far away from the shear band are still being stretched (and rotated) and their behavior dominates the trend of the averaged bond z-length for the weak bonds.

The plot of the averaged bond z-angle in Figure 7 for each bond type exhibits a curvature that is almost exactly the inverse of the corresponding bond z-length plot in Figure 6. When the bonds are stretched in the z-direction, the averaged bond z-angle decreases, that is, the bonds become more parallel to the z-axis. When the bonds are reconstructed (hence less stretched), the averaged bond z-angle increases; in other words, the bonds become less aligned to the z-direction. Therefore, the same set of messages regarding the timeline of bond stretching and bond reconstruction and the relative contributions of the different types of bonds to the strength and deformability throughout the tensile test can be extracted from Figure 7 as those discussed above based on the visualization and Figure 5 and Figure 6.

## 3. Methodology

The MD simulations in this study were performed using the popular open-source software LAMMPS (Large-scale Atomic/Molecular Massively Parallel Simulator, version: 3/3/2020) [34,35], in combination with a ternary EAM interatomic potential developed specifically for the Zr-Cu-Al system [36]. A bulk crystal of pure Cu consisting of 864,000 atoms (30 by 40 by 180 lattice units) was first melted at 1600 K under the NPT (controlled number of particles, N, pressure, P, and temperature, T) ensemble, with periodic boundary conditions in all x-, y-, and z-directions. The resulting liquid structure was used to create an initial configuration of the ternary alloy Zr_47_Cu_46_Al_7_ by random substitution of 457,920 Cu atoms with 397,440 Zr atoms and 60,480 Al atoms. The ternary system was relaxed at 1600 K for 50 ps to reach the equilibrium molten state and then cooled down to 300 K at a rate of 1 K/ps to obtain an MG sample of Zr_47_Cu_46_Al_7_. The resulting MG sample had dimensions of ~12.4, 16.5, and 74.2 nm in the x-, y-, and z-directions, respectively.

Next, the x-dimension of the sample was trimmed down to 5.5 nm by removing atoms from both sides, to approximate the overall sample aspect ratios commonly used in tensile experiments. The periodic boundary conditions in the x- and y-directions were turned off. The sample was relaxed at 300 K for 50 ps and then subjected to a constant-strain-rate (10^−4^/ps) uniaxial tensile loading along the z-direction until fracture. The sample temperature was maintained at ~300 K during the tensile test using the Nosé–Hoover thermostat. The atomic coordinates and the overall stress (pzz) on the sample were saved periodically to the dump- and thermo-files, respectively, for later analysis.

After completion of the simulated tensile test, the visualization of atoms and atomic bonds and certain data analyses (e.g., overall and partial radial distribution functions (RDFs), selection of atoms with significant atomic shear strain, and identification of their elemental types) were conducted using the OVITO (Open VIsualization TOol, version 2.9.0) software [37]. The cutoff distance values for defining various atomic bonds were taken from the valley between the first and the second peaks on the partial RDF curves for the target atomic pairs (e.g., Cu-Cu, Zr-Cu). This is discussed in more detail in Section 2.

For more detailed statistical analysis on bond lengths and bond angles, Matlab and custom scripts were utilized, where the bonding atoms were identified based on the same set of cutoff distance values as used in OVITO analysis. The coordinates of the identified bonding atoms were used to compute the 3D vectors of the bonds, which then provided the averaged bond length, bond z-length (i.e., projected length in the z-direction), and bond z-angle (i.e., the angle between the bond vectors and the z-axis) for each type of bonds as functions of the overall tensile strain.

## 4. Conclusions

Combining MD simulations with atomistic visualization and statistical analysis, we have examined the behaviors of different elements and atomic bonds in an exemplary multicomponent MG, Zr_47_Cu_46_Al_7_, during a uniaxial tensile test (load in z-direction) and related them to the contributions of the elements and atomic bonds to the material’s strength and deformability in different stages of deformation. The main findings are summarized below:

(1). In terms of elemental contributions to the material deformability, Cu and Al make bigger contributions than Zr to the localized plastic deformation (STZs) in the pre-yielding stage, as evidenced by their fractions among the large-shear-strained atoms that clearly exceed (for Cu) or nearly match (for Al) their percentages in the alloy composition; after yielding, Zr catches up in its contribution to the plastic deformation (shear band) with a fraction among the large-shear-strained atoms nearly matching (slightly below) its percentage in the alloy composition.

(2). Regarding elemental contributions to the material strength, Zr makes significant contribution to both the yield strength and post-yielding residual strength, as evidenced by its underrepresentation in the large-shear-strained atoms mentioned above as well as the strong behavior of all the Zr-related bonds (Zr-Zr, Zr-Cu, and Zr-Al) summarized below. However, Cu and Al also contribute to the material strength, through Cu-Al and especially Zr-Cu and Zr-Al bonds.

(3). All the six types of bonds existing in the material undergo stretching (bond length increment) and rotation towards the z-axis (bond z-angle decrement) initially, and then, once significant bond reconstruction occurs, the averaged bond length decreases and the averaged bond z-angle increases.

(4). The transition from stretching and rotation to reconstruction, which can be called “bond yielding”, occurs at different times for the different types of bonds.

(5). The weak bonds, Al-Al and Cu-Cu, are reconstructed shortly after the start of the tensile loading, clearly before the apparent yielding of the material, indicative of a significant contribution of the weak bonds to the localized plastic deformation (STZs) prior to yielding.

(6). The intermediate bonds, Zr-Zr and Cu-Al, and the strong bonds, Zr-Al and Zr-Cu, are reconstructed at times more coinciding with the material’s yielding, implying their significant roles in deciding the material’s yielding, yield strength, and elastic deformability.

(7). Even after the apparent yielding of the material, the strong bonds, Zr-Al and Zr-Cu, exhibit a slower drop in the averaged bond lengths and a slower rise in the averaged bond z-angle than the other bonds, suggesting that the strong bonds are the main source of residual strength and continued deformability after the material’s yielding.

These findings provide unprecedented details about the activities of the different elements and atomic bonds during deformation of a multicomponent MG, as well as their contributions to the material’s strength and deformability. As the first example of its kind, this study can be extended to other MG compositions or other classes of multicomponent alloys (e.g., high-entropy alloys) to further advance our understanding of the mechanical properties and deformation mechanisms of complex alloys and improve our ability to control them by design of composition.

## Figures and Tables

**Table 1:** Cutoff distances for different types of bonds.

Bond Type	Zr-Zr	Zr-Cu	Zr-Al	Cu-Cu	Cu-Al	Al-Al
Cutoff distance (Å)	4.1	3.7	3.9	3.2	3.3	3.4

**Table 2:** Cohesive energies for like bonds and enthalpy of mixing for unlike bonds from literature [32,33].

Al-Al	Cu-Cu	Zr-Zr	Cu-Al	Zr-Al	Zr-Cu
Cohesive energy (eV/atom)			Enthalpy of mixing (kJ/mole)		
3.39	3.49	6.25	−1	−44	−23

## Data Availability

The original contributions presented in the study are included in the article/Supplementary Materials, further inquiries can be directed to the corresponding author.

## Supplementary Materials

The following supporting information can be downloaded at: https://www.mdpi.com/article/10.3390/molecules29215005/s1. Movies S1–S6.
